# Warped linear mixed models for the genetic analysis of transformed phenotypes

**DOI:** 10.1038/ncomms5890

**Published:** 2014-09-19

**Authors:** Nicolo Fusi, Christoph Lippert, Neil D. Lawrence, Oliver Stegle

**Affiliations:** 1 eScience Group, Microsoft Research, Los Angeles, California 90024, USA; 2 Department of Computer Science, University of Sheffield, Sheffield S10 2HQ, UK; 3 European Molecular Biology Laboratory, European Bioinformatics Institute, Cambridge CB10 1SD, UK

## Abstract

Linear mixed models (LMMs) are a powerful and established tool for studying genotype–phenotype relationships. A limitation of the LMM is that the model assumes Gaussian distributed residuals, a requirement that rarely holds in practice. Violations of this assumption can lead to false conclusions and loss in power. To mitigate this problem, it is common practice to pre-process the phenotypic values to make them as Gaussian as possible, for instance by applying logarithmic or other nonlinear transformations. Unfortunately, different phenotypes require different transformations, and choosing an appropriate transformation is challenging and subjective. Here we present an extension of the LMM that estimates an optimal transformation from the observed data. In simulations and applications to real data from human, mouse and yeast, we show that using transformations inferred by our model increases power in genome-wide association studies and increases the accuracy of heritability estimation and phenotype prediction.

## Abstract (web summary)

Linear mixed models (LMMs) provide a powerful method for studying genotype–phenotype associations. Here the authors present a LMM application that estimates an optimal transformation from observed data and increases the accuracy of heritability estimation and phenotype prediction.

## Graphical abstract

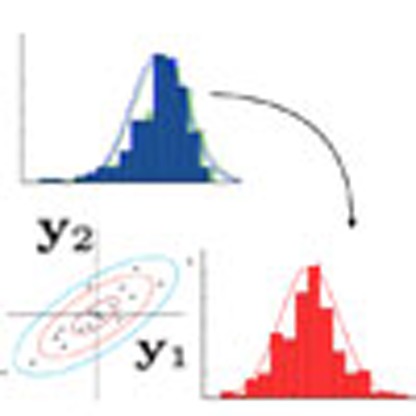

Linear mixed models (LMMs) are widely used in genetic studies of quantitative traits in humans and model organisms. This family of models is attractive because in addition to modelling the effect of individual genetic variants, the LMM effectively accounts for polygenic effects and confounding because of population structure or family relatedness. Important applications of LMMs include genome-wide association studies (GWASs)^1^,^2^, narrow-sense heritability estimation^3^,^4^ and phenotype prediction^5^,^6^,^7^,^8^.

One of the core assumptions of LMMs is that the residual noise is Gaussian distributed, and deviations from Gaussianity can result in model misspecification^9^. To mitigate this problem, it is a common practice to apply transformations to phenotypes such that their marginal distributions are approximately Gaussian. For instance, if the scale of the phenotype spans several orders of magnitude, a log-transformation may be used as a preprocessing step to then perform genetic analyses on the transformed values. Log transformations have also been used when the phenotypic measurement is defined as the ratio between a foreground and a background signal, such as in gene expression measurements from microarrays^10^ or when analysing composite phenotypes (for example, the ratio between total cholesterol and high-density lipoprotein (HDL)). Nonetheless, the set of transformations that are being used in genetic studies is not limited to the canonical log transformation^11^,^12^,^13^,^14^ and no single transformation can be considered a universal solution. For instance, a recent study of 58 different mouse traits^15^ considered a semi-manual selection procedure to identify an appropriate phenotype transformation for each single trait. In this context, manual selection of transformations has two drawbacks. First, there is no established criterion to select one transformation over another; in particular, naïve comparison of the model likelihood is not applicable for this task (see Methods). This is because the objective is not to obtain Gaussian distributed phenotypes, but rather Gaussian distributed residuals after fitting an unknown genetic model. Moreover, the number of possible transformations that can be manually explored is limited. Exhaustively, testing large numbers of alternative transformations, each characterized by a different parameterization, is time consuming and can result in a multiple hypothesis testing problem, for example, if power in GWAS is used as a selection criterion.

Here we investigate the practical relevance of phenotype transformations in the context of key applications of LMMs in genetics. We propose the warped linear mixed model (WarpedLMM), a principled generalization of the standard LMM that allows to fit phenotype transformations while performing genetic analyses. We show how the likelihood principle can be extended to objectively assess alternative transformations in the light of the observed genotype and phenotype data. WarpedLMM can seamlessly be used in place of traditional LMMs, and it identifies transformations that are both parametric and invertible, thus permitting to predict phenotypic values on the original scale. This is not straightforward, for instance, when considering non-parametric transformations based on rank statistics (see Results).

We investigate the practical utility of WarpedLMM in different genetic analyses, where we consider both extensive simulation studies, as well as real data from human, mouse and yeast. We compare WarpedLMM to established preprocessing approaches for phenotypes, such as Box-Cox transformations^16^ or rank transformations^17^, in combination with a standard LMM, demonstrating that WarpedLMM more accurately recovers the true underlying transformations. Our results show that WarpedLMM can be used as an effective replacement of the standard LMM in a wide range of genetic analyses, resulting in an increase of power in GWAS, a reduction of bias in narrow-sense heritability estimation and improved phenotype prediction accuracy. In particular, in a GWAS on four metabolic traits from the Northern Finland Birth Cohort, WarpedLMM identified four additional associations that were not found when using a standard LMM on untransformed phenotypes.

## Results

### Summary of the method

Both, when specifying a phenotype transformation or when inferring it from the data (for example, using WarpedLMM), the implicit assumption is that the quantitative trait under genetic control is unobserved or latent, with the observed phenotype being determined by a nonlinear mapping *g* that links the latent phenotype to the observed measurements (Supplementary Fig. 1). Thus, to recover the true genetic model, an estimate of the ideal phenotype transformation *f* (where *f*=**g**^−1^) is needed. If we denote the observed phenotype for individual *n* as *y*_*n*_, an estimate of the latent phenotype *z*_*n*_ can be obtained by applying the function *f*, optionally parameterized by **ψ**:


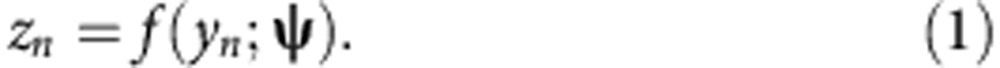


In WarpedLMM, these functions are constrained to be invertible and are termed ‘warping functions’. The functional form of ***f*** is determined by parameters **ψ**, which are inferred jointly with the remaining model parameters of the LMM. The most probable transformation can then be inferred by maximizing the sum of the standard log likelihood and a Jacobian term that accounts for the complexity of the fitted warping function. Several functional forms of the warping functions can be chosen (see Methods), differing in number of free parameters and in the complexity of the functions they can represent. In the following, we consider a particular family of functions initially proposed by Snelson *et al.*^18^, which can be expressed as linear combination of a linear scaling term and multiple nonlinear step functions. If the observed phenotype *y*_*n*_ does not require a transformation, only the linear term will be used. Otherwise, the function will consist of both the linear term and one or more step functions.

### Simulations

First, we considered the problem of narrow-sense heritability estimation on simulated data, where ground truth is available. We simulated phenotypic effects based on genotype data from the HapMap project^19^, performing multiple simulations while varying the proportion of variance explained by the genotype, the number of simulated causal variants and the sample size of the simulated data set. In each experiment, we first simulated phenotype values from a linear additive genetic model (see Methods), and then applied a nonlinear function *g* (see Supplementary Fig. 1), yielding the final observed phenotype. In an effort to keep our simulations as realistic as possible, we considered a set of transformations that have previously been identified in the genetic analysis of a diverse set of global quantitative traits in mouse^15^. In the following, we choose the function *g* to be a variant of an exponential function, such that the ideal phenotype transformation is a log transformation. Analogous results for alternative functions are shown in Supplementary Figs 2 and 3.

In addition to considering alternative genetic models, we considered smooth interpolations of the warping function, linearly interpolating between the identity function (no transformation) and a completely nonlinear function (full transformation). We then compared the ability of the WarpedLMM and the LMM to estimate the true simulated heritability from the transformed phenotypes. We also considered an LMM applied to phenotypes pre-processed using a log transformation (Log-LMM) and a transformation fit using the Box-Cox method (Box-CoxLMM), both of which are commonly used in practice^16^,^20^,^21^,^22^,^23^,^24^.

**Figure 1:**
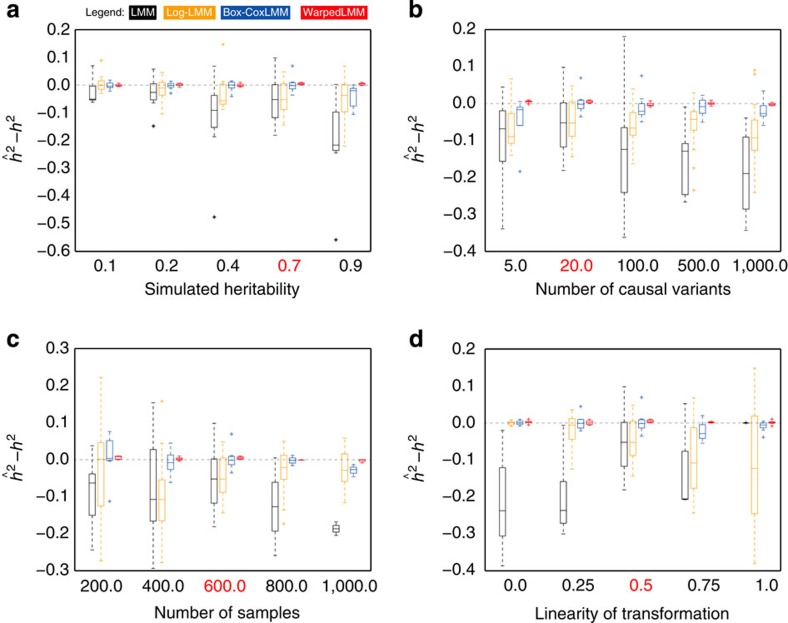
Simulation experiment considering variants of an exponential transformation as true phenotype transformation and comparing different LMM approaches for estimating the genetic proportion of phenotype variability (narrow-sense heritability, *h*^2^). (**a**) Changing the simulated heritability (**b**) considering different numbers of causal variants (**c**) increasing the sample size and (**d**) decreasing the nonlinearity of the true simulated transformation (a value of 1 correspond to a linear function, whereas 0 denotes a fully nonlinear function. See Methods for details). When varying each individual parameter, the remaining simulation settings remained constant with the default parameters being highlighted in red. Heritability estimates were obtained using WarpedLMM, a standard LMM, an LMM on log transformed phenotype data and an LMM on Box-Cox preprocessed phenotypes. We repeated this simulation procedure 50,000 times in order to have a sufficiently large sample size to investigate all the regimes described above.

When comparing the heritability estimates to the true simulated heritability, WarpedLMM consistently was more accurate than all the other methods, whereas the LMM tended to underestimate the heritability. In the most extreme cases, the LMM estimates had a downward bias of up to 30%, whereas WarpedLMM was close to unbiased (less than 1%). The overall accuracy of WarpedLMM for heritability estimation was remarkably robust to changes of the simulation parameters, including the simulated heritability level (Fig. 1a), the number of causal variants (Fig. 1b), the number of samples (Fig. 1c) or the strength of the nonlinear transformation (Fig. 1d). Strikingly, we also observed that the estimation bias of the standard LMM persisted even in the regime of large sample sizes (Fig. 1c). Similarly, we found that the accuracy of heritability estimates using an LMM deteriorated when increasing the true simulated heritability (Fig. 1a) or the number of causal variants (Fig. 1b). Not surprisingly, the degree of nonlinearity of the transformation had the strongest effect on the model accuracy (Fig. 1d), where even subtle nonlinearity of the transformation functions markedly affected the heritability estimates. It should be noted that, even in settings where the true transformation function was a linear function (rightmost point in Fig. 1d), WarpedLMM achieved approximately the same estimation error as a standard LMM, demonstrating that the method is robust and can be safely applied even in settings where no transformation is needed. Interestingly, pre-processing the data using a log transformation (Log-LMM) only worked well if the true underlying transformation was completely nonlinear (leftmost point in Fig. 1d) and deviations from complete nonlinearity resulted in progressively more biased estimates. Additional comparisons, considering alternative classes of transformations and methods, are shown in Supplementary Figs 2 and 3. These comparisons include a simpler variant of WarpedLMM that does not include individual genetic factors with large effects, showing how the joint modelling approach taken in WarpedLMM (see Methods) greatly improve accuracy in the recovery of the true underlying transformation. We have also considered other commonly used transformations (log and squared root), finding that usage of a rigid *a priori* defined set of pre-processing transformations can induce significant biases in the heritability estimates.

### Mouse data from Valdar *et al*

**Figure 2:**
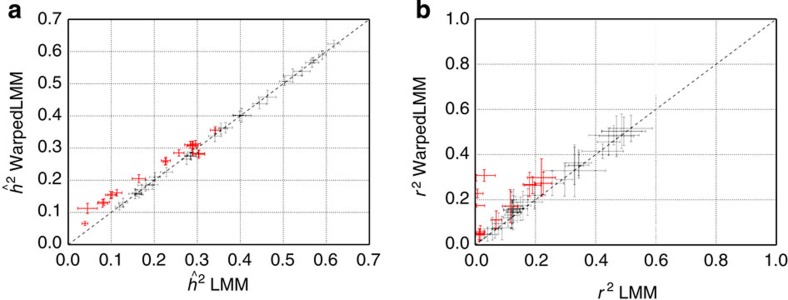
Comparative analysis of WarpedLMM and a LMM for 58 phenotypes in the mouse data set. (**a**) Heritability estimates using a LMM on the untransformed phenotype versus the heritability estimates obtained by WarpedLMM. Empirical error bars were obtained from ten bootstrap replicates, using 90% of the data in each replicate. Significant differences are coloured in red (paired *t*-test, *α*=0.05). (**b**) Out-of-sample prediction accuracy assessed by the squared correlation coefficient *r*^2^, considering either a LMM on the untransformed data or a WarpedLMM. Prediction accuracies were assessed from ten random train-test splits. Phenotypes with significant deviations in prediction accuracy of the LMM and the WarpedLMM are highlighted in red (paired *t*-test, *P*-value≤0.05).

Next, we revisited data from a heritability study in a structured mouse population^15^. This study highlighted that the careful definition of a specific transformation for each phenotype studied is important for accurate quantitative trait loci (QTL) mapping. Although this process was guided by an initial Box-Cox fit, the authors performed additional manual tuning of the resulting function for each one of the 58 phenotypes. Here, we compared the heritability estimates obtained using a standard LMM on untransformed phenotypes with those obtained from WarpedLMM. Covariates such as age, gender, body weight, litter number and cage density were included as fixed effects in both models. For 18 of the 47 phenotypes, the two models yielded significantly different heritability estimates (Fig. 2a, *P*-value ≤0.05 from a paired *t*-test). In the majority of these cases (17 out of 18), WarpedLMM yielded higher heritability estimates than the standard LMM (up to threefold), again showing that the choice of phenotypic transformation can significantly affect heritability estimates.

Unlike in the simulated experiments described in the previous section, we lack an accurate gold standard to validate the heritability estimates on real data. To this end, we assessed the consistency of our findings by comparing both models in an out-of-sample prediction task. We performed a tenfold cross-validation experiment, where each model was repeatedly trained on 90% of the data to predict the phenotype from genotype on the remaining 10% of the samples. WarpedLMM was consistently more accurate in out-of-sample predictions than a standard LMM (Fig. 2b), even for phenotypes where the corresponding heritability estimates of the WarpedLMM model were lower than those from the standard LMM (Supplementary Fig. 6b). This suggests that the phenotype transformations recovered by WarpedLMM can help avoiding under- or overfitting in applications of LMMs. This confirms our results on simulated data and gives confidence that the heritability estimates of WarpedLMM are also more accurate on real data.

Finally, when comparing the transformations identified by WarpedLMM to those manually derived by Valdar *et al.*^15^, we found that the functions estimated by WarpedLMM were consistently in the same functional category (linear, logarithmic and so on) as those reported in the original study, however, with slight differences in parameterization (Supplementary Fig. 4).

Supplementary Figs 5a,b and 6a provide equivalent results for a similar study in a yeast cross^25^, demonstrating that these findings hold also for other systems.

### WarpedLMM for GWAS

In addition to heritability estimation and prediction, WarpedLMM can also be used to perform GWASs. To test this, we revisited genotype and phenotype data from the Northern Finland birth cohort^26^ where we analysed four related metabolic traits: HDL, low-density lipoprotein (LDL), triglycerides and C-reactive protein (CRP). This selection of four phenotypes is particularly interesting, because although the phenotypes are closely related in biological mechanism, the primary analysis^26^ of these data was performed using logarithmic transformation for two of the four phenotypes (triglyceride, CRP), whereas the remaining phenotypes (HDL, LDL) were analysed on the linear scale.

**Figure 3:**
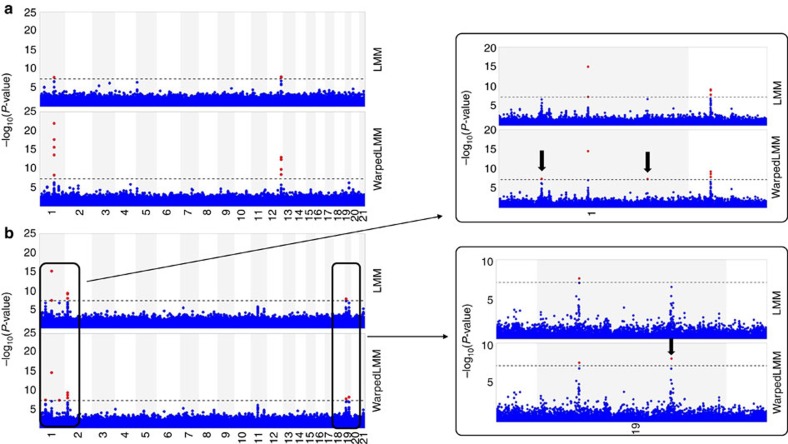
Manhattan plots comparing a standard LMM to a WarpedLMM in a GWAS of two metabolic traits in the NFBC1966 study. (**a**) The GWAS results for C-reactive protein, and (**b**) the GWAS results for low-density lipoprotein. Red circles denote significant associations (*α*<5 × 10^−8^, marked on the plots with a dashed line). The two rightmost panels show an enlarged view of interesting regions in chromosomes 1 and 19, with black arrows highlighting loci that were identified only when using WarpedLMM.

Here, we compared the results of a univariate GWAS using three different methods: WarpedLMM, an LMM applied to untransformed phenotypes^1^ and an LMM on phenotypes transformed as reported in the original paper^26^. Association results from all methods were appropriately controlled for type 1 error rate (genomic control for all methods was 1.00±0.01). Overall, WarpedLMM yielded increased GWAS power to detect associations (Supplementary Table 1). For example, WarpedLMM identified a total of six distinct QTL (*P*-value ≤5 × 10^−8^) for LDL cholesterol levels (Fig. 3b), whereas the naïve LMM only identified three out of these six. Notably, two of the three additional associations detected by WarpedLMM have previously been implicated with LDL. In particular, rs4844614 has been significantly associated with LDL in an analysis of the same data using linear regression^26^ (omitting correction for population structure) and rs4844614 has been identified in a large meta-analysis^27^.

Likewise for HDL, WarpedLMM identified three QTLs, whereas both alternative methods missed one of these associations. Even in settings where WarpedLMM did not yield novel associations, such as in the analysis of CRP, the model yielded greatly increased sensitivity such that known association signals did stand out to a greater extent (Fig. 3a).

We also found that applying WarpedLMM to fit a separate warping functions for each of the four phenotypes, led to an increase of pairwise (Pearson) correlations between these phenotypes, which can be important for multivariate genetic analyses with linear Gaussian models^28^,^29^ (Supplementary Fig. 7). Similar increases in correlation coefficients can be obtained by semi-parametric transformations, which have previously been proposed as preprocessing step for multivariate analyses^17^ on the same data set. Unlike WarpedLMM, this approach is based on rank-standardizing transformations of individual phenotypes before regressing out covariates, followed by an additional rank-standardization step^17^. This procedure implicitly assumes that contributions from genotype and covariates are independent and that the overall genetic effect is small and hence genotype can be ignored when determining the phenotype transformation. Although these assumptions may be violated in other settings, comparative analysis with transformations fit by WarpedLMM confirmed that the semi-parametric approach proposed by Zhou and Stephens is appropriate for these data^17^. Indeed, we found striking correlations between the functions recovered (Supplementary Fig. 8) by both methods and the respective *P*-values under these transformations in the context of a single trait GWAS on each trait (*ρ*=0.99±0.01 for −log_10_ *pv*, Supplementary Fig. 9).

Finally, we evaluated the genetic model fit by the WarpedLMM and compared it to a standard LMM using out-of-sample phenotype prediction. As the warping functions fit by WarpedLMM are invertible, we can assess the prediction accuracy of a genetic model on the natural scale of the raw phenotypic values, which is not feasible when using rank-based preprocessing methods^17^. Whereas the heritability estimates from WarpedLMM were either increasing or decreasing compared with a standard LMM, depending on the trait (Supplementary Table 2), the out sample correlation coefficients were consistently higher for WarpedLMM (Supplementary Table 3). Again, this suggests that WarpedLMM more accurately explains the true genetic component of phenotypic variability. Overall, these experiments give confidence that WarpedLMM can be applied as a robust preprocessing procedure for GWAS.

## Discussion

Although preprocessing methods are widely used in practice to approximately identify and invert an unknown phenotype transformation^11^,^12^,^13^,^14^,^17^,^20^,^22^,^23^,^24^,^30^,^31^, so far there has been no principled approach to assess and fit these transformations while accounting for genetic information and covariates.

Here we have shown how the classical LMM can be extended to estimate phenotype transformations directly from the data. Our experiments show that WarpedLMM is able to significantly improve accuracy and power in key genetic analyses and that unsuitable phenotype transformations can lead to profound analysis biases. Although an important application of WarpedLMM is the identification of phenotype transformation to improve downstream analysis, we emphasize that the model is more than an *ad-hoc* preprocessing procedure. The objective function of the model can be derived from first principles, resulting in an extension of the mixed model that accounts for both the data likelihood and the complexity of the fitted transformation (see Methods). As a result, our approach can be directly applied to tasks commonly tackled using LMMs, such as GWAS, heritability estimation and phenotype prediction.

When applying WarpedLMM to studies in mouse and yeast, we found that the model tended to increase the estimates of heritability. Although in a minority of traits the heritability estimates decreased, we note that the model consistently improved out-of-sample prediction. This shows that inappropriate phenotype transformations can lead to biased heritability estimates and overfitting, an effect that has previously been reported by others^32^. Remarkably, although WarpedLMM has a larger number of parameters than a standard mixed model, the model did not overfit even when considering sample sizes that are much smaller than the ones used in typical studies (Fig. 1a).

Although we have focused on some of the most established tasks in genetic analysis, WarpedLMM can easily be adapted to more specialized tasks. For example, it is straightforward to use the model in combination with multi-locus mixed models^33^ or mixed models that jointly consider multiple phenotypes^28^,^29^. WarpedLMM finds the transformation function while jointly taking into account all the available covariates, polygenic genetic background and individual genetic loci with large effect sizes. This joint approach helps to ensure that the model residuals are Gaussian distributed, rather than the phenotype itself. The importance of this principle has been recognized in previous work^17^, where the authors employed a three-step procedure, which consisted of rank transforming the phenotype, regressing out the covariates and rank transforming the residuals again. This approach assumes that the genotype explains only a small portion of the variance and hence ‘Gaussianizing’ phenotype data on the null model is valid. Although this approach is reasonable in some settings, deviations from this assumption remain a concern^31^. This highlights the need for more principled approaches such as WarpedLMM, putting the principles phenotype transformations that leverage additional information from covariates and genetic data on solid statistical grounds.

Finally, we note that there may be settings where WarpedLMM does not achieve optimal results. Similar to other existing methods, the model estimates a transformation under the assumption that the noise level in the transformed phenotype space is constant. This assumption may be violated in some cases such as when dealing with count data or binary phenotypes. In such instances, it will remain appropriate to use generalized LMMs with non-Gaussian likelihoods that incorporate stronger assumptions about the nature of the data^34^. Nonetheless, the number of phenotypes being measured is constantly increasing and only a small fraction will respect the well-defined properties of canonical link functions that are commonly used in generalized LMMs. In these instances, the advantages of the WarpedLMM model are clear: it allows for robust analyses of a broad spectrum of phenotypes without the need to carry out manual exploration of suitable transformations.

## Methods

### The warpedLMM

We model the observed non-normal distributed phenotype *y*_*n*_ of each individual *n* with an unobserved normal distributed phenotype *z*_*n*_ that results from transforming *y*_*n*_ using a monotonic function *f* with some parameters **ψ**.


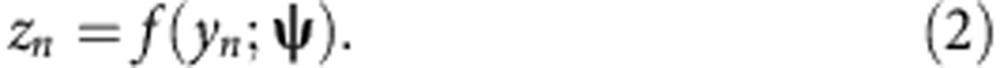


The generative model for the normal distributed phenotype *z*_*n*_ can then be written as


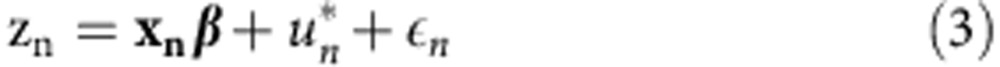


where **x**_**n**_ holds the covariates for individual *n*, ***β*** are fixed effects, 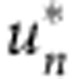 denotes a random effect that captures the polygenic genetic effect from S* loci and ε_*n*_ is independent normal distributed noise.

Given this LMM, the likelihood for *N*-by-1 vector **z**=*f* (**y**;**ψ**) of transformed phenotypes for a sample of *N* individuals follows as


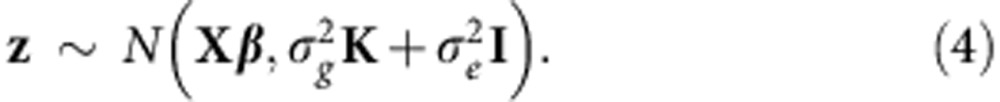


Here, **K** denotes the genomic relatedness matrix^35^ computed from all S genotyped single-nucleotide polymorphisms (SNPs), pre-processed to have zero mean and unit variance and stored in the *N* × *S* matrix **G**:


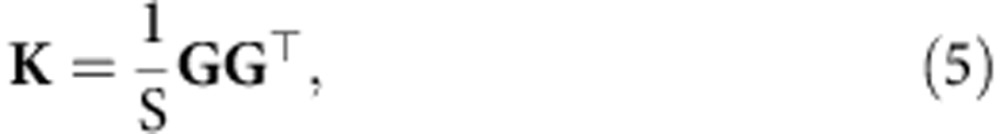


while 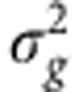 is the total amount of genetic variance and 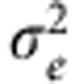 is the error noise variance.

### Choosing a monotonic warping function

Instead of specifying a predefined static transformation, WarpedLMM identifies the most probable transformation 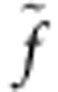 for a given data set by maximizing the likelihood (4) with respect to the model parameters and the parameters of the warping function. Several types of warping functions can be chosen in principle, for example, differing in the number of free parameters that must be inferred and in the complexity of the function that can be represented.

Throughout this paper, we use the warping function first proposed by Snelson *et al*.^18^, who proposed a similar model in a context outside of genetics, and choose the transformation for the phenotype *y*_*n*_ of each sample as


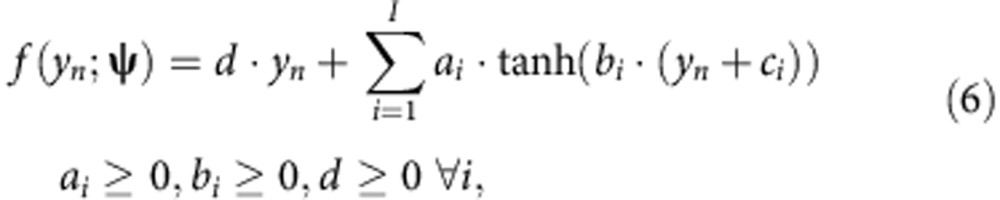


where **ψ**=(*d*,*a*_1_,*b*_1_,*c*_1,_...,*a*_*I*_,*b*_*I*_,*c*_*I*_).

In this parameterization, *f* is a sum over *I* nonlinear step functions, where the parameter *a*_*i*_ controls the step size, *b*_*i*_ controls the steepness and *c*_*i*_ determines the location. Finally, the parameter *d* denotes the slope for the linear part (in *y*_*n*_) of the function. The only parameter that requires manual specification is the number of step functions *I*. We followed the recommendation in Snelson *et al.* and used *I*=3 step functions for all of our experiments. This specific choice appears to be remarkably robust and effective across a variety of experiments.

In principle, any parametric monotonic function can be used in place of the function suggested above. For instance, a warping function based on the popular Box and Cox^16^ transformation could be used as an alternative:


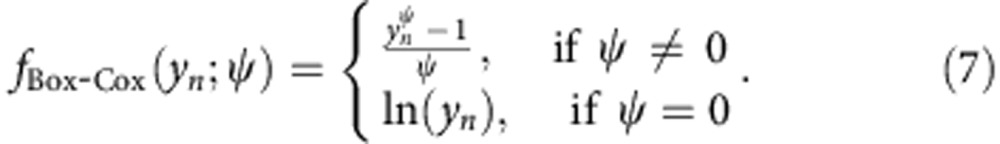


This classical warping function is controlled by a single parameter, and thus can be useful when the large number of parameters of the function proposed above is a concern. Other types of warping functions include shifted logarithmic transformations or shifted and scaled arsinh functions, which have been proposed in the context of variance stabilizing transformations for microarray data^36^,^37^. Again, all of these transformations can be expressed in the framework of the WarpedLMM.

### Parameter estimation

The model parameters (
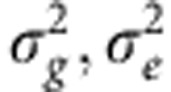
) and the parameters of the warping function (**ψ**) are estimated by maximizing a form of LMM likelihood. By taking the logarithm of equation (4), the negative log likelihood *L* for the hidden normal distributed phenotype **z** is obtained as


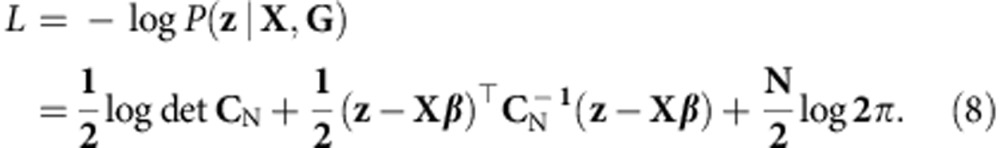


The previous equation is not accounting for the fact that **z** is really a transformation of the observed phenotype **y**. This transformation can be taken into account by including the corresponding Jacobian term, yielding an extended log likelihood for **y** as


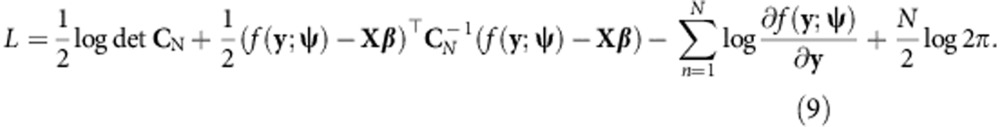


It is then possible to fit the model by minimizing equation (9) with respect to the parameters of the LMM and the transformation.

### Incorporating strong genetic effects

Although the realized relationship matrix **K** can accurately capture the relatedness between individuals in the presence of many causal variants with small effect sizes, it does not necessarily do so when the genetic signal is mostly due to a small number of causal variants. To address this setting, several approaches^33^,^38^,^39^ have been proposed to select large effects for inclusion in the model. Here we perform a forward selection procedure^38^,^39^, iteratively including in the model variance components that capture individual loci with large effects. Of course, alternatives^40^ to the forward selection technique described here could be used to select the genetic variants to be included in the model.

At iteration *t*, the conditional distribution of the latent phenotype **z** follows as


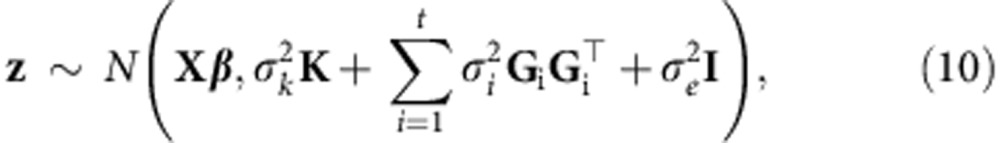


where the parameters 
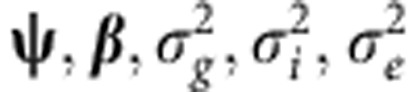
 are re-estimated at each iteration.

In each iteration, the SNP with the strongest individual effect is determined by fixed effects testing^2^ of all genetic markers against the current transformed phenotype ***z***_*t*_ using the current set of variance components as the relatedness matrix. A marker is selected if its *q*-value^41^ is smaller than a threshold, which we set to 0.05 for all our experiments. This algorithm converges when no marker achieves genome-wide significance at the FDR level specified.

The genetic effects incorporated in the model at the end of this procedure can in general be beneficial for certain tasks such as phenotype prediction. Here we only use them to better reconstruct the transformation function, and we do not take them into account while doing prediction or heritability estimation. Finally, it is important to notice that we model these individual genetic variants as random effects, placing a Gaussian prior over their effect sizes and integrating them out. If the number of selected genetic markers is small, they can alternatively be modelled as fixed effect covariates, for example, using restricted maximum likelihood.

### Phenotype prediction

The fitted WarpedLMM model can also be used to predict the unobserved phenotype of a new individual indexed by * given the genotype alone. Assuming a fully observed sample of *N* individuals, we can use the parameter estimates under model (4) to compute the best linear unbiased predictor 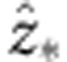 of the new individual’s phenotype on the normal distributed scale


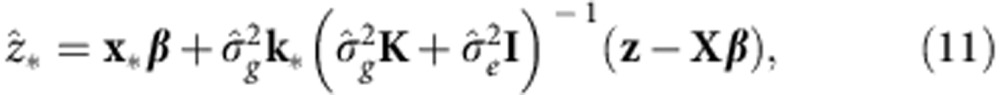


where x_*_ is a vector of covariates for the new individual, k_*_ is a 1-by-*N* vector that contains the genomic relatedness between the new individual and all the individuals in the original sample.

To get an estimate of the phenotype on the original scale, we apply the reverse transformation *f*^*−*1^ to the best linear unbiased predictor


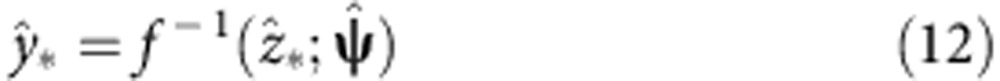


The reverse transformation *f*^−1^ is obtained by numerically inverting *f* using Newton-Raphson updates, as previously proposed by Snelson *et al.*

### Estimating heritability

It is possible to obtain an estimate of the narrow-sense heritability *h*^2^ in the normal distributed scale by computing a chip heritability 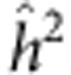 from common genotyped markers in the LMM (4).


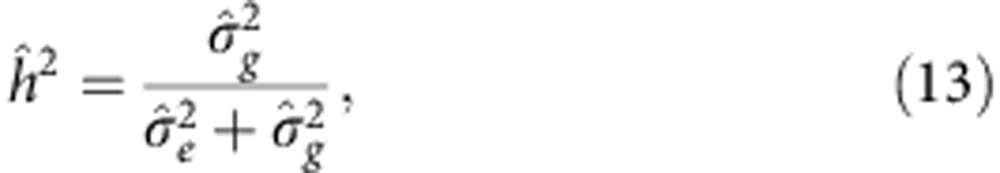


where 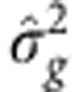 and 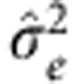 are restricted maximum likelihood estimates of 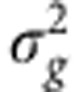 and 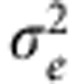.

### Simulation study

The simulated data are generated taking genotypes from hapmap3 (ref. 19) chromosome 22 and sampling from a standard LMM with additive genetic effects and Gaussian distributed noise. In each simulation, we sample *h*^2^ from {0.1,0.20,0.40,0.70,0.9}, the number of causal variants from {5,20,100,500,1,000}, the number of samples from {200,400,600,800,1,000} and the variance explained by covariates from {0.0,0.25,0.5,0.70,0.9}. We can then recover the noise level conditioned on *h*^2^, and the covariates variance.

Finally, we pick a transformation *f*(*y*) from the set of transformations used in Valdar *et al.*^15^ For the experiments in the main paper, we consider exp(*y*); results for alternative transformations are presented as Supplementary Material. We then transform the phenotype as *z*=*t*·*y*+(1−*t*) *f (y)*, where *t* is a parameter that determines the intensity of the transformation and is sampled from {0.0, 0.25, 0.5, 0.75, 1.0}. We repeated this simulation procedure 50,000 times in order to have a sufficiently large sample size to investigate all the regimes described above.

### Mouse data

We used mouse data from Valdar *et al.*^15^ This data set contains between 1,700 and 1,940 samples (depending on phenotype missingness), 10,132 markers and 47 phenotypes.

### Yeast data

We used yeast data from Bloom *et al.*^25^ This data set contains 1,008 samples, 11,623 markers and 46 phenotypes.

### Human data

We used the data from Sabatti *et al.*^26^ and applied the same filtering criteria described in Zhou and Stephens^17^. This resulted in 5,255 individuals and 328,517 SNPs.

### Software

An implementation of WarpedLMM is available at http://github.com/pmbio/warpedLMM.

## Author contributions

O.S. and N.F. conceived the method. N.F., O.S. and C.L. designed the experiments. N.F. performed the experiments. N.F. and O.S. analysed the data. N.D.L., N.F., O.S. and C.L. contributed computational tools. O.S., N.F. and C.L. wrote the paper.

## Additional information

**How to cite this article:** Fusi, N. *et al.* Warped linear mixed models for the genetic analysis of transformed phenotypes. *Nat. Commun.* 5:4890 doi: 10.1038/ncomms5890 (2014).

## Supplementary Material

Supplementary Information Supplementary Figures 1-9, Supplementary Tables 1-3 and Supplementary References
