# Supplementary material for: Warped linear mixed models for the genetic analysis of transformed phenotypes
Source: Nat Commun. 2014 Sep 19;5:4890. doi: 10.1038/ncomms5890 (PMC4199105; doi:10.1038/ncomms5890)
Supplement: Supplementary Information — Supplementary Figures 1-9, Supplementary Tables 1-3 and Supplementary References [file ncomms5890-s1.pdf]

Supplementary Information

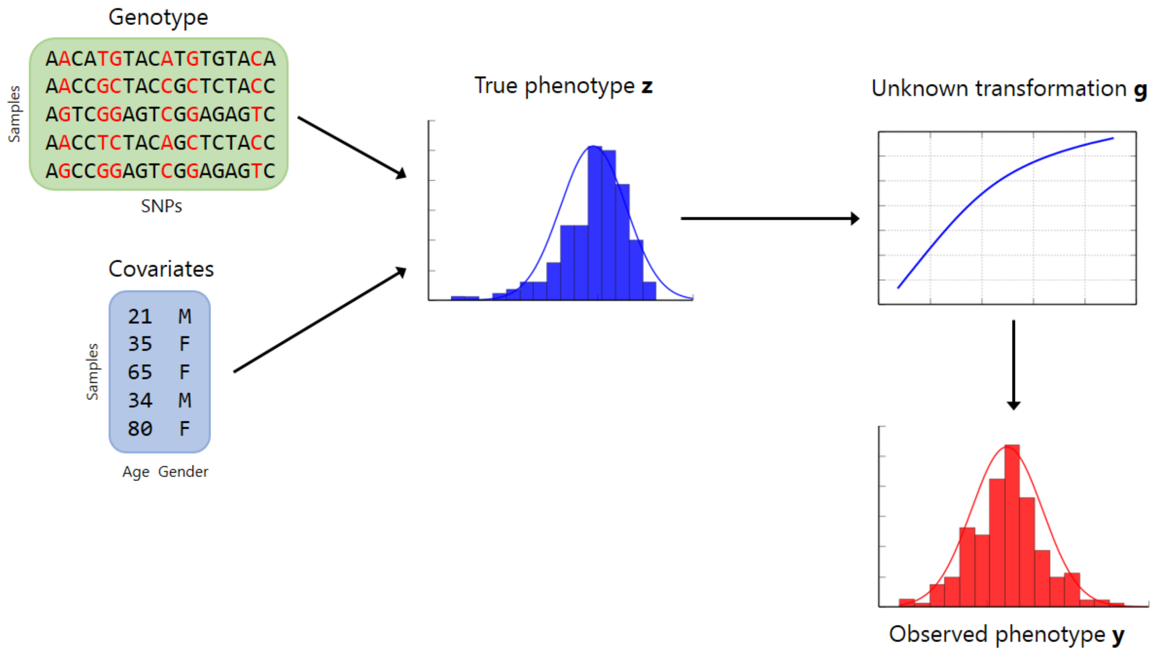

Supplementary Figure 1 Illustration of the assumptions underlying the WarpedLMM. The genetic model of interest determines the latent (true) phenotype profiles **z** (blue histogram). The measured phenotype data **y** (red histogram) are then derived from **z** via an unknown transformation **g**. The goal of WarpedLMM and phenotype transformations in general is to recover the latent phenotype z by estimating the inverse transformation of g.

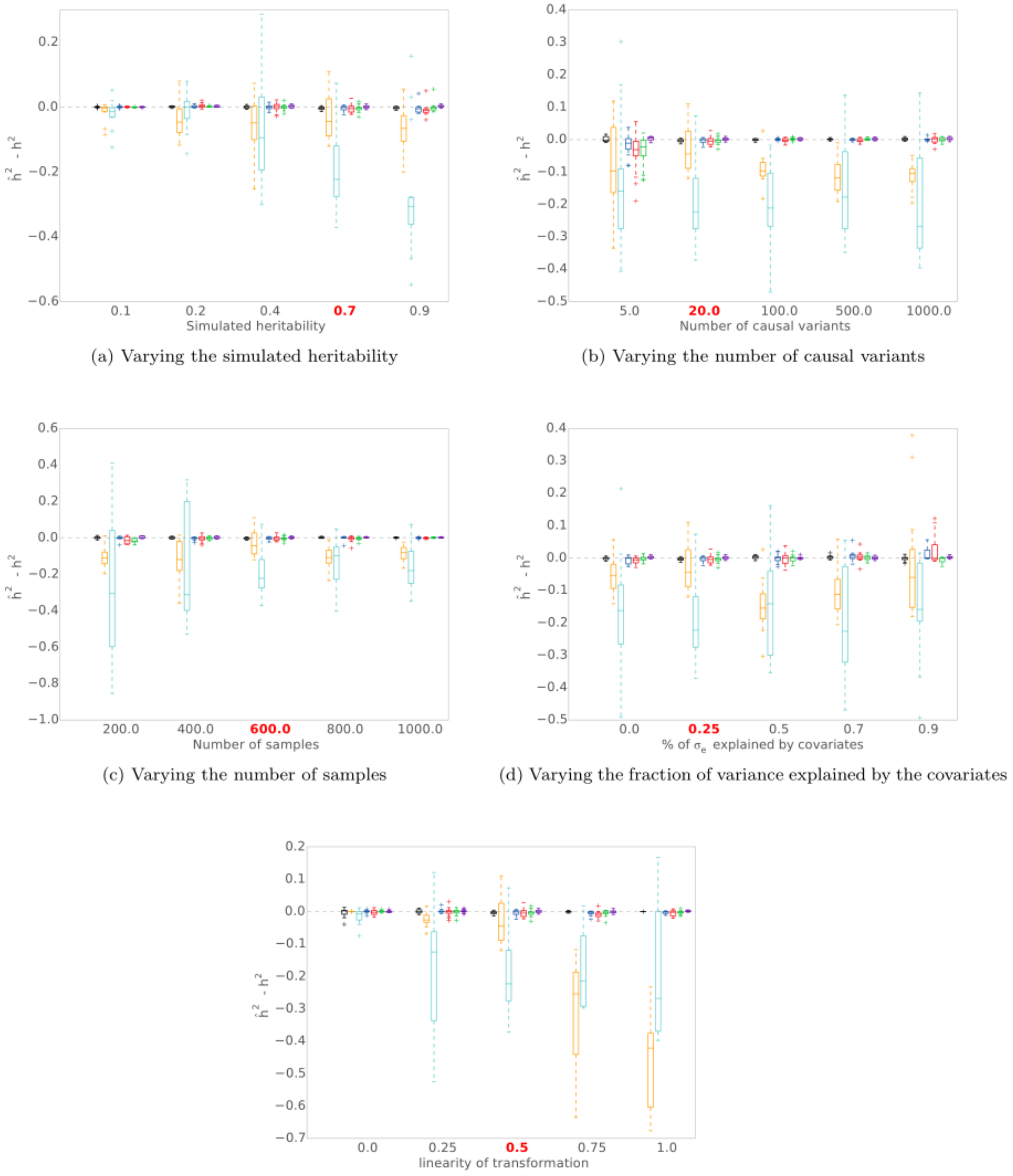

(a) Varying the simulated heritability

(b) Varying the number of causal variants

(c) Varying the number of samples

(d) Varying the fraction of variance explained by the covariates

(e) Varying the intensity of the transformation

Supplementary Figure 2 Comparison of alternative linear mixed-model approaches for estimating the genetic contribution to phenotype variability (narrow sense heritability h^2). As done in the main paper, we evaluate the difference between the estimated and the true genetic variance across 50,000 simulated experiments. In this particular experiment we considered a different transformation ($z = \sqrt{y}$) and included comparisons to a rank-based transformation and a simpler version of the WarpedLMM model where strong genetic effects are not included in the model. Legend: **LMM**, **Ideal transformation** ($y = z^2$), **log transformation**, **Box-Cox**, **Rank transformation**, **WarpedLMM without large-effect loci**, **WarpedLMM**.

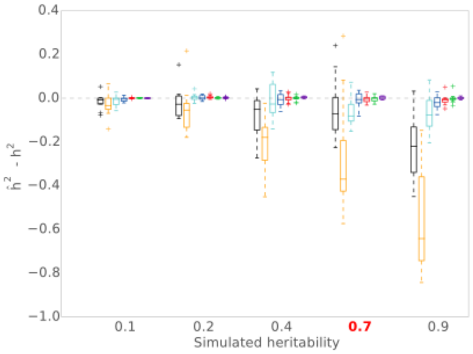

(a) Varying the simulated heritability

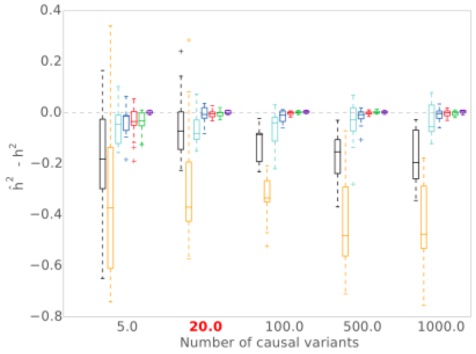

(b) Varying the number of causal variants

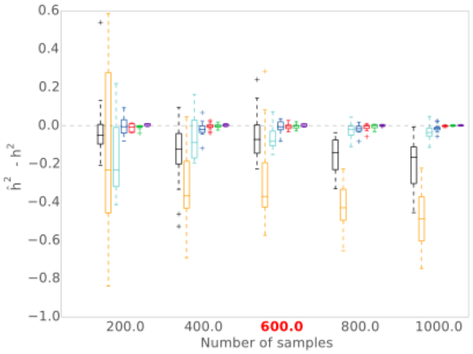

(c) Varying the number of samples

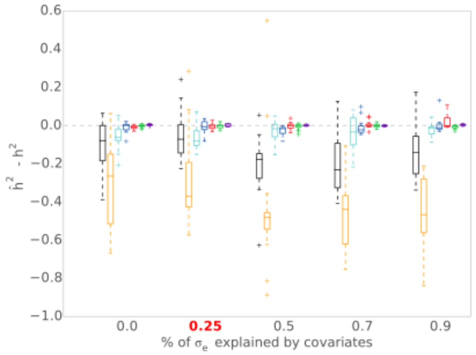

(d) Varying the fraction of variance explained by the covariates

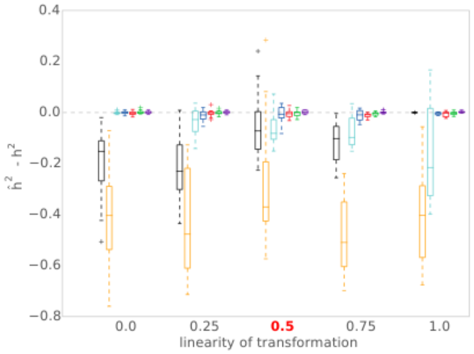

(e) Varying the intensity of the transformation

Supplementary Figure 3 Comparison of alternative linear mixed-model approaches for estimating the genetic contribution to phenotype variability (narrow sense heritability, h^2). As done in the main paper, we evaluate the difference between the estimated and the true genetic variance across 50,000 simulated experiments. Here, we considered the transformation $z = exp(y)$ and included comparisons to a rank-based transformation and a simpler version of the WarpedLMM model where strong genetic effects are not included in the model. Legend: **LMM**, **$y = z^2$ transformation**, **ideal transformation** ($y = log(z)$), **Box-Cox**, **Rank transformation**, **WarpedLMM without large-effect loci**, **WarpedLMM**.

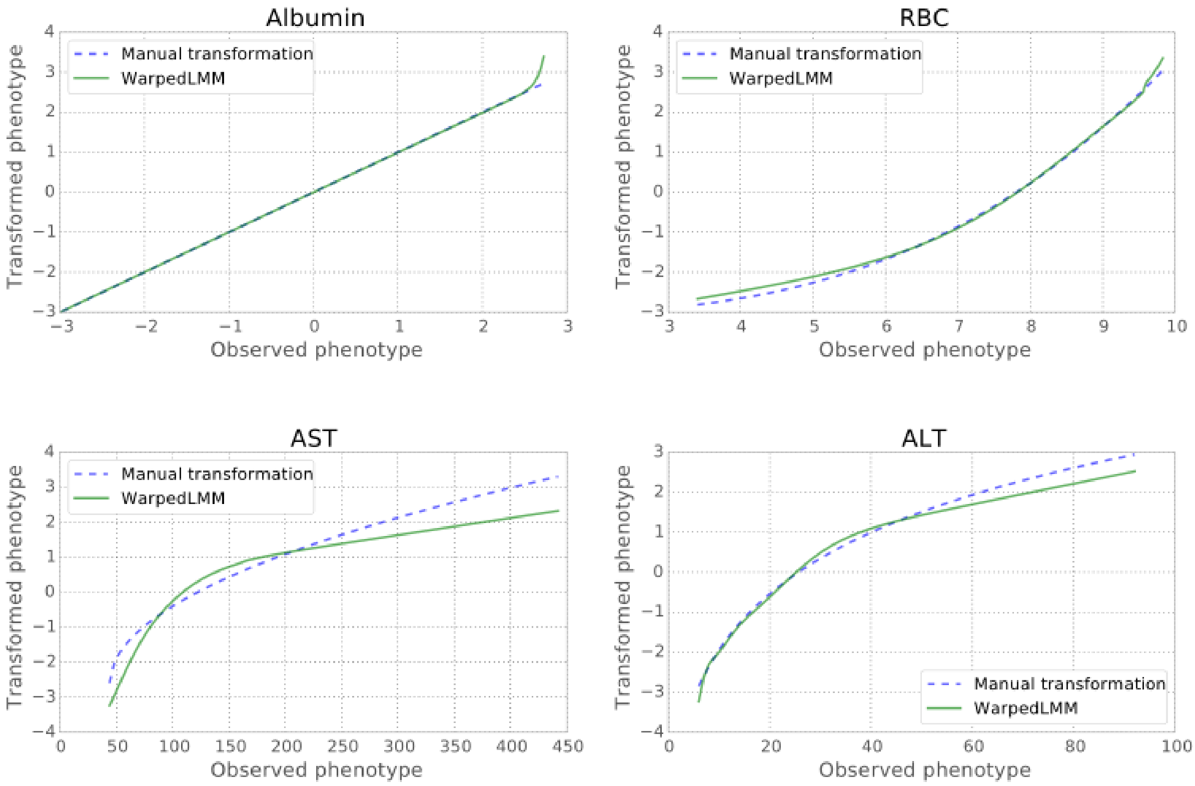

Supplementary Figure 4 Comparison of the manual transformations reported in Valdar et al.[1] and the transformations found by WarpedLMM for 4 representative phenotypes of the the mouse dataset.

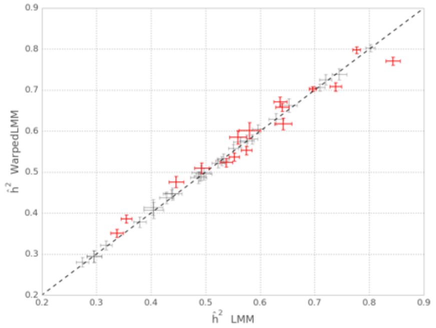

(a) LMM $\hat{h}^2$ vs WarpedLMM $\hat{h}^2$

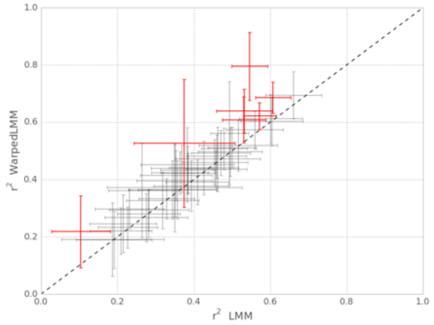

(b) LMM r^2 vs WarpedLMM r^2

Supplementary Figure 5 Heritability estimates and out-of-sample prediction accuracy for 46 phenotypes in an F2 yeast cross [2]. The experimental analysis and setup are analogous to the results reported on mouse in the main paper. Panel (**a**) shows heritability estimates using a LMM on the untransformed phenotype versus the heritability estimates obtained by WarpedLMM. Empirical error bars were obtained from 10 bootstrap replicates, using 90 % of the data in each replicate. Significant differences are colored in red (paired t-test, $\alpha = 0.05$). These methods results in significantly deviating heritability estimates (paired t-test, $\alpha = 0.05$) for 17 phenotypes (38%), most of which with increased heritability by WarpedLMM compared to the standard approach (11 of 17, 65%). Panel (**b**) shows out-of-sample prediction accuracy assessed by the squared correlation coefficient r^2, considering either a LMM on the untransformed data and a WarpedLMM. Prediction accuracies were assessed from 10 random train-test splits. Phenotypes with significant deviations in prediction accuracy of the LMM and the WarpedLMM are highlighted in red (paired t-test, p-value ≤ 0.05). As for the mouse experiments in the main paper, WarpedLMM model consistently yielded improved prediction accuracy, irrespective of whether the heritability estimate increased or decreased compared to a standard LMM (Supplementary Figure 6a). Overall, these results suggest that even phenotypes obtained in highly controlled settings tend to be transformed, leading to both overestimation and underestimation of the narrow-sense heritability.

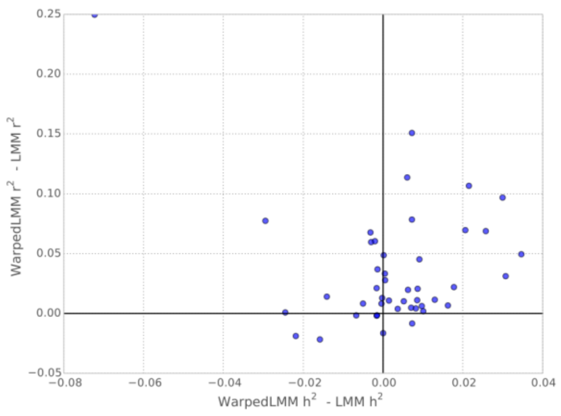

(a) Difference in $\hat{h}^2$ vs difference in r^2 in the yeast dataset

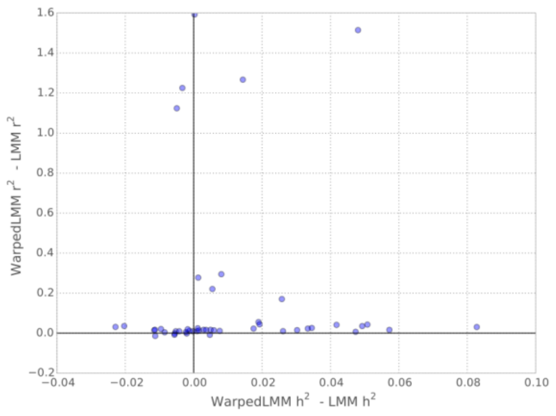

(b) Difference in $\hat{h}^2$ vs difference in r^2 in the mouse dataset

Supplementary Figure 6 Comparison of the difference in heritability estimation and the out-of-sample prediction performance in (**a**) the yeast dataset (**b**) the mouse dataset. These results show that, on both datasets, WarpedLMM tends to improved prediction accuracy, irrespective of whether the estimated heritability increases or decreases.

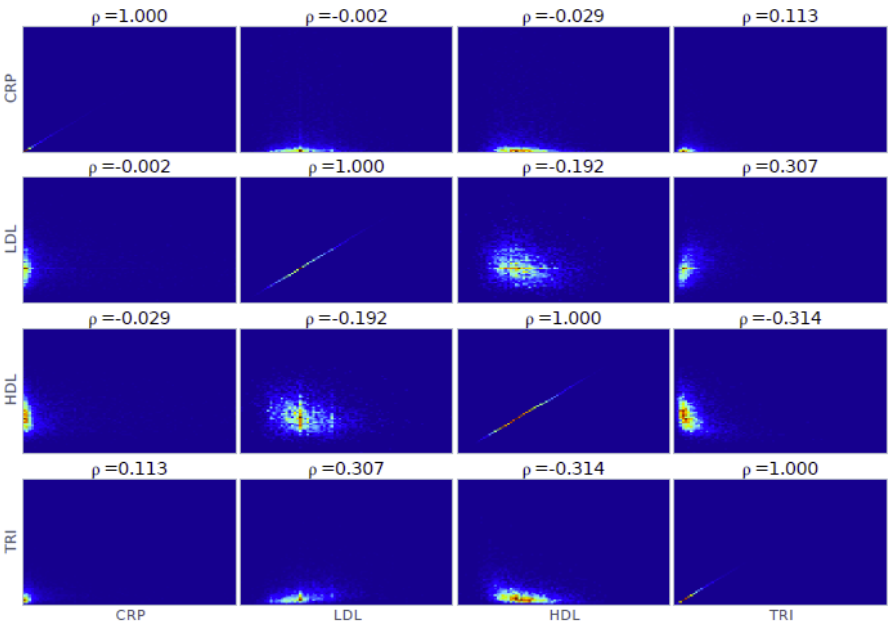

(a) Without transforming the phenotypes

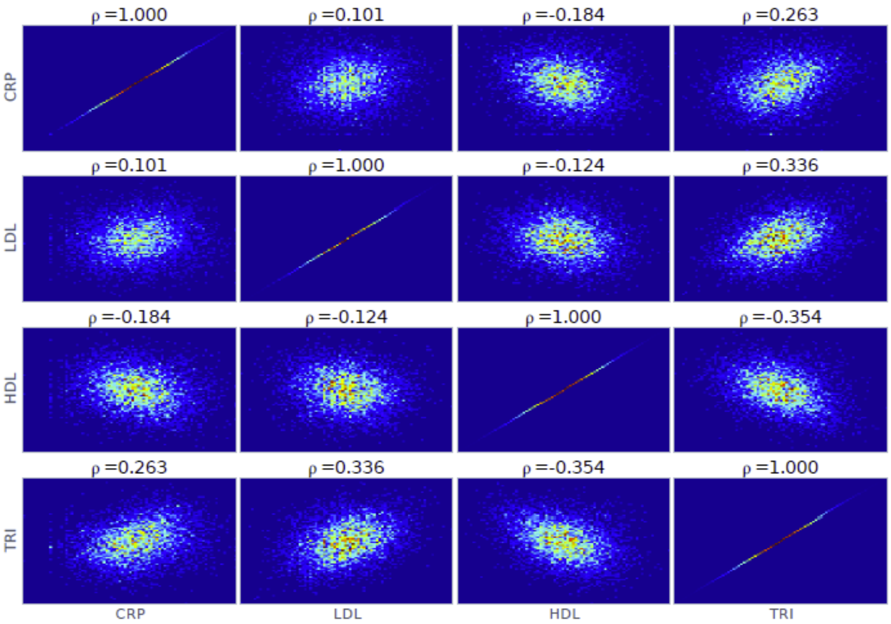

(b) Applying the transformation found by WarpedLMM

Supplementary Figure 7 Correlation analysis between the 4 phenotypes (C-reactive protein, low-density lipoprotein, high-density lipoprotein, triglycerides) considered in Zhou and Stephens[3] (a) without transforming the phenotypes (b) after applying the transformation reconstructed by WarpedLMM. Shown are Pearson correlation coefficients, which similar to a multi-trait mixed model are affected by the scale of the phenotypes. Note that because WarpedLMM transformations are monotonic, Spearman rank correlations are invariant under this type of transformations.

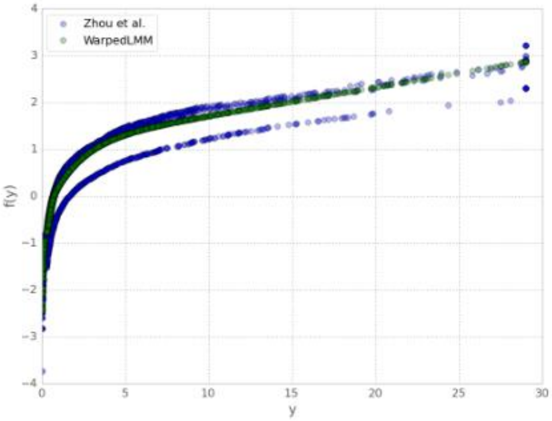

(a) CRP

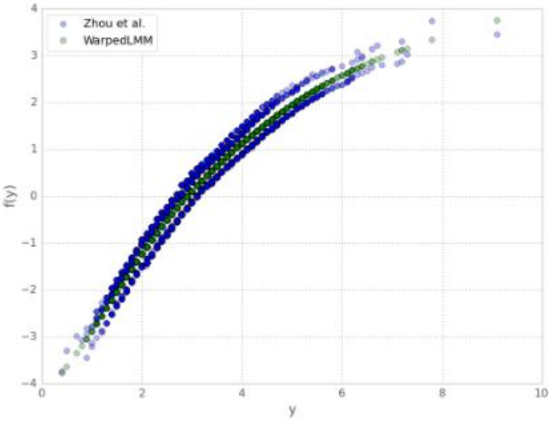

(b) LDL

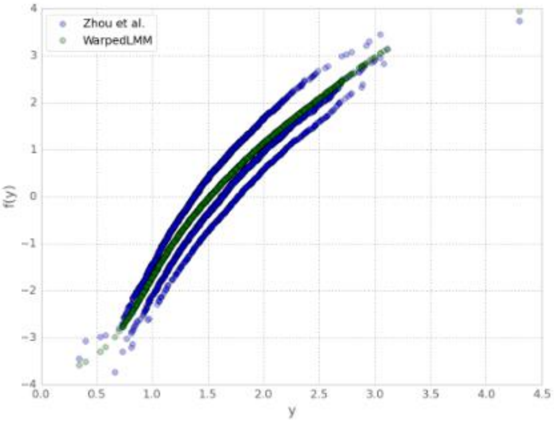

(c) HDL

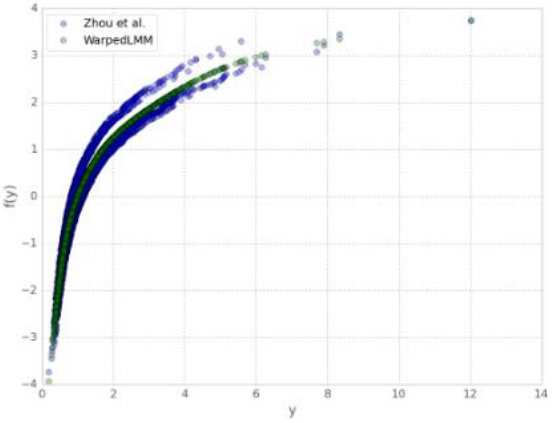

(d) TRI

Supplementary Figure 8 Comparison of the rank transformations reported in Zhou and Stephens[3] and the transformations found by WarpedLMM on the human dataset. The multiple blue lines for the rank transformation are due to the intermediate step in the method by Zhou and Stephens[3], where covariates are regressed out. For this reason, in the case of the rank transformation, the y-axis shows $f(y - \boldsymbol{X}\beta)$, where $\boldsymbol{X}$ is a matrix containing the covariates. Despite this difference, this figure shows that WarpedLMM and the approach proposed in [3] yield transformations in the same functional class.

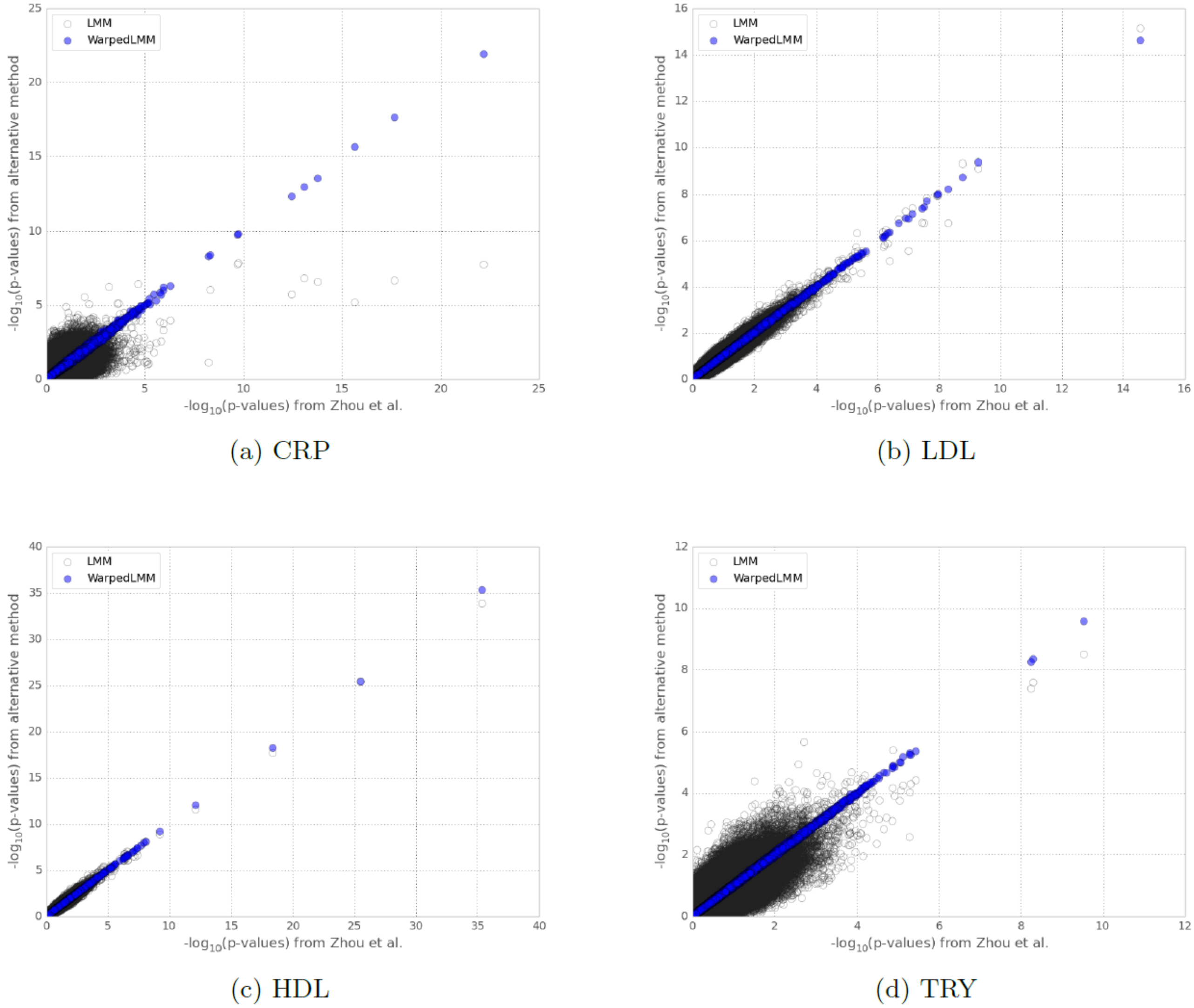

Supplementary Figure 9 Comparison of p-values obtained from a parametric rank transformation regressing out covariates [3] and WarpedLMM. The plots show the $-\,log_{10}\,pv$ of the method described by Zhou and Stephens on the x-axis versus the $-\,log_{10}\,pv$ obtained when using warpedLMM (solid blue circles) and a LMM (empty black circles). All methods considered yielded well-calibrated p-values with genomic controls of 1.00 ± 0.01.

Supplementary Table 1 Association results for the human dataset. Significantly associated loci (at significance level 5×10^{-8}) have a green background, while non-significant ones are colored in red.

	Chr	Position	WarpedLMM	LMM on untransformed	LMM using transformation from original paper [4,5]
**CRP**	1	(157908973, 157966663)	1.24e-22	1.81e-08	2.74e-22
	12	(11987334, 119923227)	1.04e-13	1.46e-08	3.34e-12
**LDL**	1	55579053	3.63e-08	1.81e-07	1.81e-07
	1	109620053	2.44e-15	7.34e-16	7.34e-16
	1	205941798	4.21e-08	1.74e-07	1.74e-07
	2	(21085700, 21165196)	4.41e-10	8.05e-10	8.05e-10
	19	11056030	1.99e-08	1.49e-08	1.49e-08
	19	50087106	6.14e-9	1.81e-07	1.81e-07
**HDL**	15	(56470658, 56478046)	9.62e-13	2.78e-12	2.78e-12
	16	(55542640, 55564091)	4.96e-36	1.44e-34	1.44e-34
	16	(66229305, 66582496)	8.11e-09	9.79e-09	9.79e-09
	20	42475778	3.80e-08	2.49e-07	2.49e-07
**TRY**	2	(27584444, 27594741)	2.66e-10	3.15e-09	2.66e-10
	8	19875201	5.57e-09	4.08e-08	5.57e-09

Supplementary Table 2 Narrow-sense heritability estimates for the human dataset (NFBC). Standard deviations were obtained from 10 bootstrap replicates, considered 90 % of the data in each replicate.

	WarpedLMM	LMM on untransformed
**CRP**	0.15 ± 0.07	0.21 ± 0.14
**LDL**	0.20 ± 0.13	0.18 ± 0.06
**HDL**	0.21 ± 0.10	0.24 ± 0.17
**TRY**	0.16 ± 0.06	0.16 ± 0.06
**BMI**	0.17 ± 0.03	0.18 ± 0.03
**HEIGHT**	0.35 ± 0.03	0.33 ± 0.01

Supplementary Table 3 Out of sample r^2computed over 10 random train/test splits on the human dataset (NFBC)., training on 90% of the data and testing on 10%. Shown are the averages of sample correlation coefficients and standard error computed over different test sets.

	WarpedLMM	LMM on untransformed
**CRP**	0.06 ± 0.02	0.035 ± 0.01
**LDL**	0.05 ± 0.02	0.04 ± 0.02
**HDL**	0.14 ± 0.04	0.13 ± 0.03
**TRY**	0.08 ± 0.03	0.02 ± 0.02

Supplementary References

1. Valdar, W. *et al.* Genetic and environmental effects on complex traits in mice. *Genetics* **174,** 959–84 (2006).

2. Bloom, J. S., Ehrenreich, I. M., Loo, W. T., Lite, T.-L. V. & Kruglyak, L. Finding the sources of missing heritability in a yeast cross. *Nature* **494,** 234–7 (2013).

3. Zhou, X. & Stephens, M. Efficient Algorithms for Multivariate Linear Mixed Models in Genome-wide Association Studies. *arXiv Prepr. arXiv1305.4366* 1–35 (2013).

4. Sabatti, C. *et al.* Genome-wide association analysis of metabolic traits in a birth cohort from a founder population. *Nat. Genet.* **41,** 35–46 (2009).

5. Kang, H. M. *et al.* Variance component model to account for sample structure in genome-wide association studies. *Nat. Genet.* **42,** 348–354 (2010).
